# DYADIC FACTORS THAT ASSOCIATE WITH INSOMNIA IN CAREGIVERS OF PERSONS LIVING WITH DEMENTIA

**DOI:** 10.1093/geroni/igz038.1341

**Published:** 2019-11-08

**Authors:** Glenna S Brewster, Donald Bliwise, Fayron Epps, Kate Yeager, Ken Hepburn

**Affiliations:** 1 Emory University, Nell Hodgson Woodruff School of Nursing, Atlanta, Georgia, United States; 2 Emory University School of Medicine, Atlanta, Georgia, United States; 3 Georgia State University, Atlanta, Georgia, United States; 4 Emory University, Atlanta, Georgia, United States

## Abstract

Insomnia is prevalent in caregivers of persons living with dementia (PLWD); however, more research is needed to identify which dyadic factors most impact caregiver sleep. This study aimed to identify the factors associated with caregiver insomnia in the baseline component of a randomized clinical trial. A linear regression was conducted with caregiver variables (e.g., depression), and PLWD variables (e.g., disruptive nighttime behaviors) as independent variables in relation to insomnia, as assessed with Insomnia Severity Index (ISI). Caregivers (n=49) were on average 63 years, mostly female (65.3%), White (69.4%), and spouses (65.3%). Mean ISI was 6.8, indicating mild-to-moderately disturbed sleep. Multiple linear regression (F(11,32) = 13.4, p<.001) showed that both caregiver-based measures (depression, p<.023) and PLWD-based measures (disruptive nighttime behaviors, p<.001) were independently associated with ISI. Ideas about needed multicomponent dyadic interventions which target both PLWD nighttime behaviors, caregiver depression, and sleep disturbances will be discussed.

